# Missed bronchial web in a 4‐year‐old boy with foreign body aspiration: A case report

**DOI:** 10.1002/ccr3.6765

**Published:** 2023-02-03

**Authors:** Mahsa Mirzandeh del, Atefeh Abedini, Fatemehsadat Rahimi, Siamak Afaghi, Arda Kiani

**Affiliations:** ^1^ Pediatric Respiratory Disease Research Center, National Research Institute of Tuberculosis and Lung Diseases (NRITLD) Shahid Beheshti University of Medical Sciences Tehran Iran; ^2^ Chronic Respiratory Diseases Research Center, National Research Institute of Tuberculosis and Lung Diseases, Masih Daneshvari Hospital Shahid Beheshti University of Medical Sciences Tehran Iran; ^3^ Prevention of Metabolic Disorders Research Center Research Institute of Endocrine Sciences, Shahid Beheshti University of Medical Sciences Tehran Iran

**Keywords:** bronchial stenosis, bronchoscopy, chest 3D‐CT, congenital tracheal stenosis, virtual bronchoscopy

## Abstract

Congenital bronchial webs are extremely rare and usually remain undiagnosed due to nonspecific symptoms. Herein, we reported a 4‐year‐old case of the bronchial web who was initially undiagnosed upon bronchoscopy following foreign body aspiration and afterward misdiagnosed as childhood asthma through his consistent cough and exertional dyspnea for several months.

## Graphical abstract

Bronchial webs mainly remained undiagnosed or misdiagnosed due to nonspecific symptoms. This diagnosis should be considered in childhood persistent cough or dyspnea, specifically in cases not responding to conventional anti‐asthma medications.

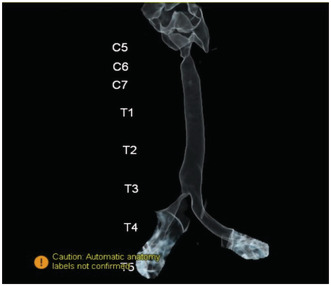

## 1 INTRODUCTION

Bronchial stenosis could be either acquired (following trauma, infection, inflammation, and autoimmune disorders) or congenital due to bronchial webs though its incidence is extremely scarce.^1^ Congenital bronchial webs are often undiagnosed during infancy and misdiagnosed as childhood asthma or foreign body aspiration later as they are not presented with specific manifestations. Patients mainly complain of shortness of breath, especially during exertion, persistent cough, wheezing, or even infections.^2^ The true incidence of the bronchial web is not precisely known as they may go unrecognized throughout life. It is estimated that 1 in 10,000 births could be afflicted with bronchial webs.^3^ Lung computed tomography (CT) scan could be helpful for screening for airway anomalies. Moreover, bronchoscopy is both diagnostic and a therapeutic option. Herein, we described a 4‐year‐old boy with a right bronchial web who had been primarily diagnosed with a case of foreign body aspiration and afterward treated as approaching childhood asthma.

## 2 CASE REPORT

**FIGURE 1:**
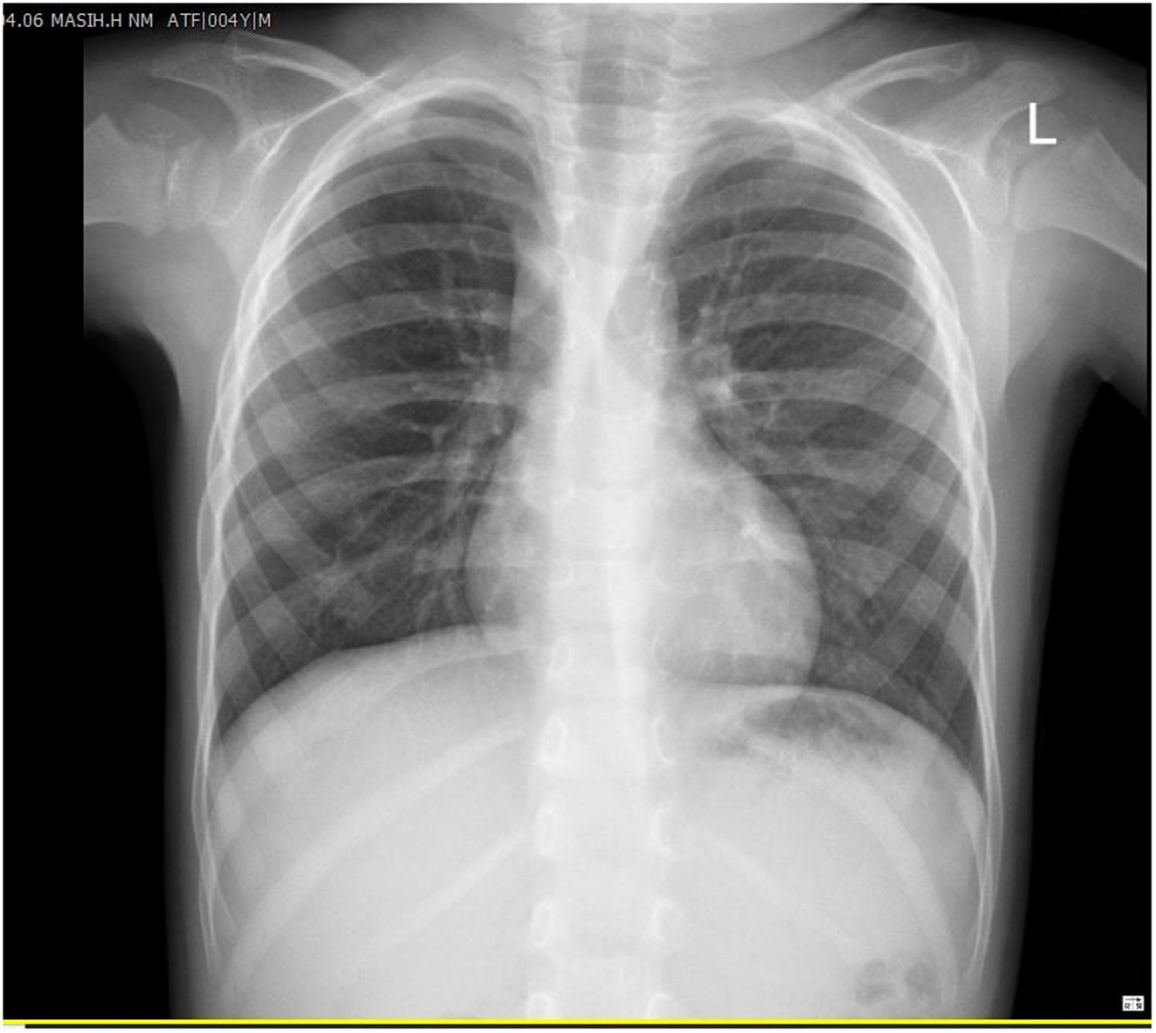
Normal chest x‐ray upon admission

**FIGURE 2:**
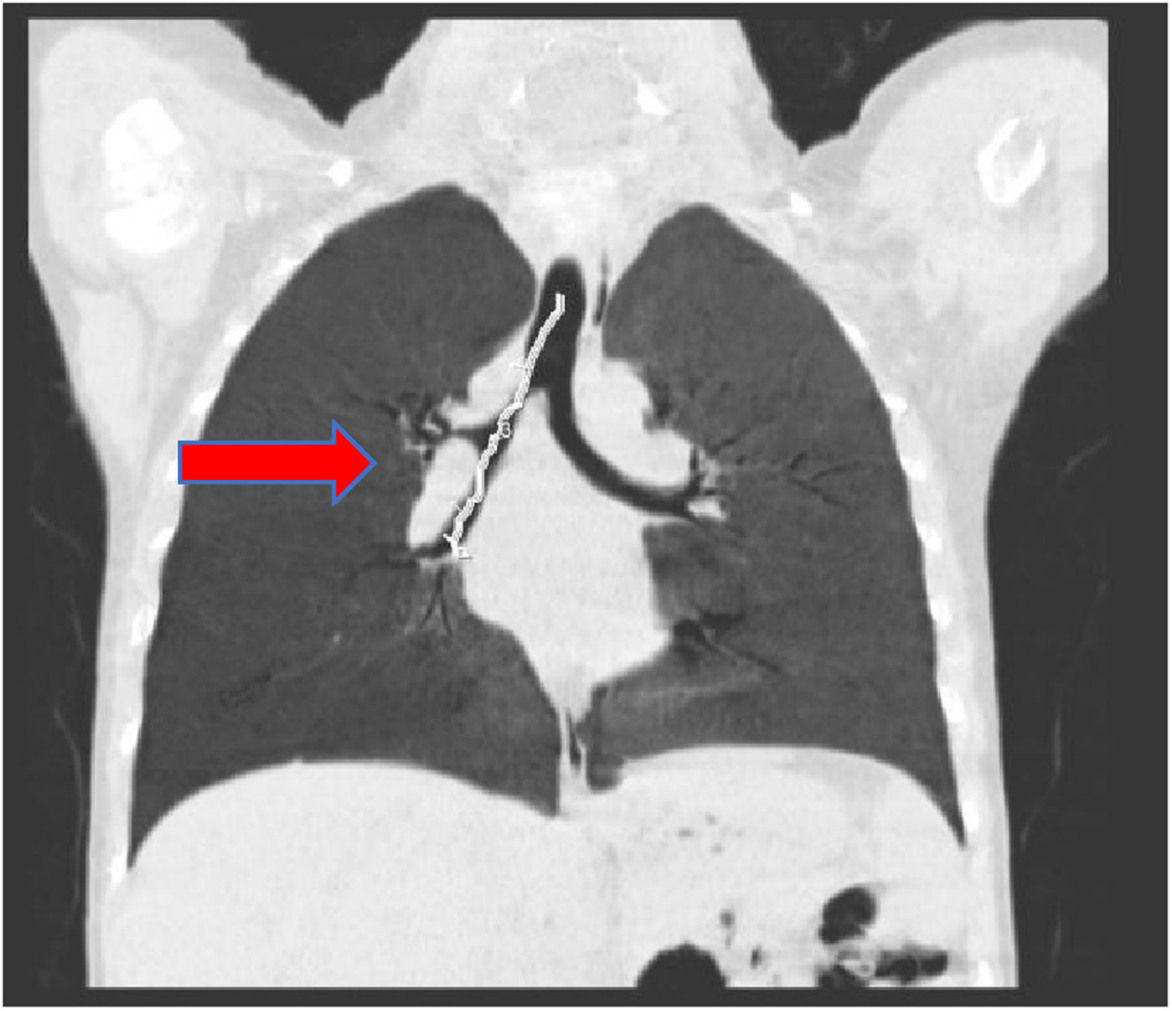
Partial obstruction of right main bronchus is remarked in the coronal cut of the chest CT scan

**FIGURE 3:**
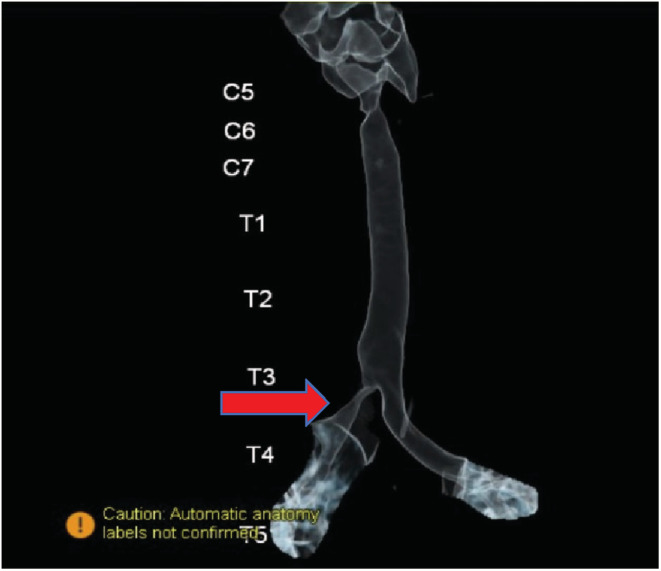
Partial narrowing of right main bronchus at the levels of third thoracic in the 3‐dimensional chest CT scan (virtual bronchoscopy)

**FIGURE 4:**
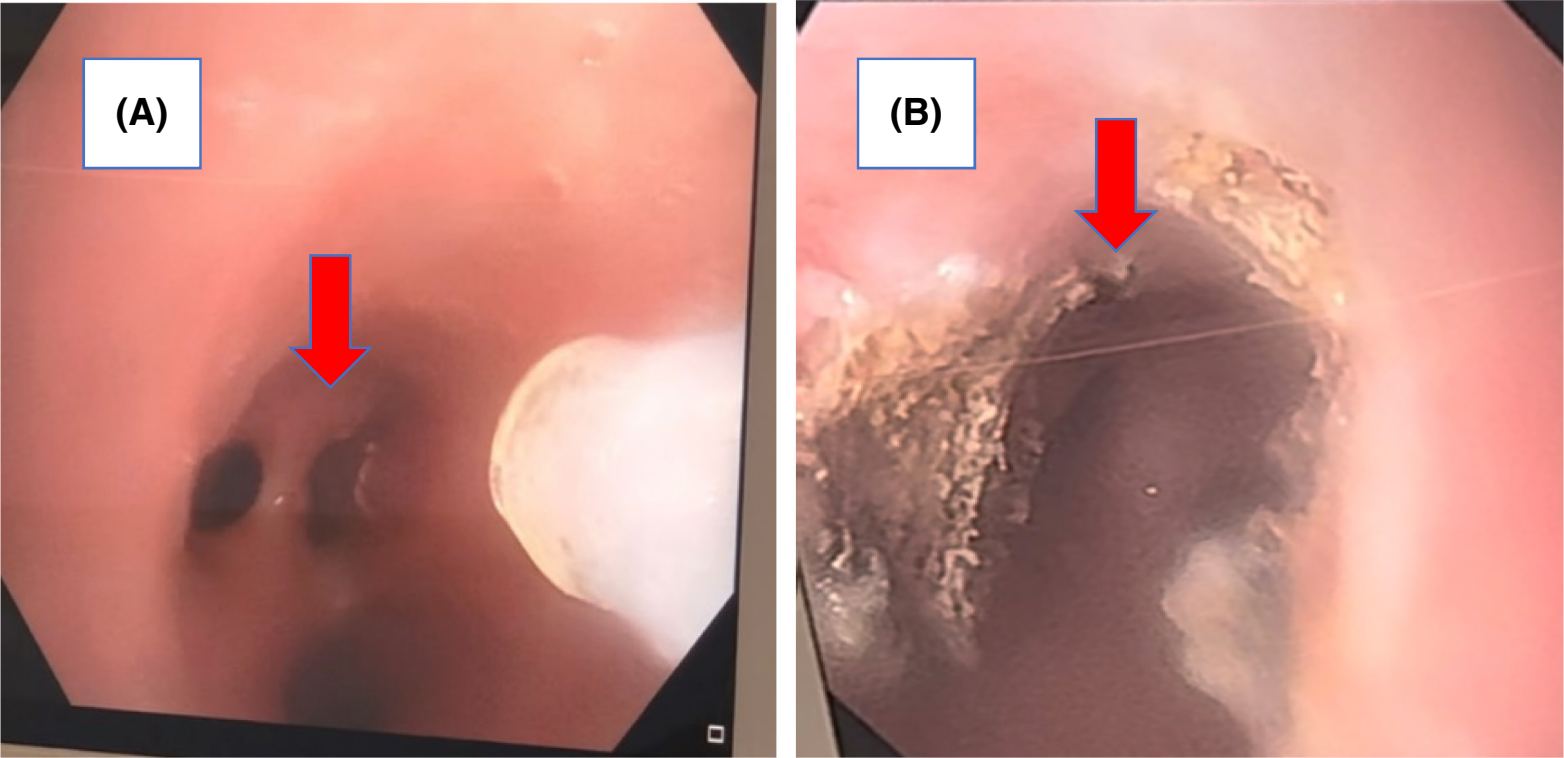
Bronchoscopy in a case of bronchial web in right main bronchus. (A) Partial stenosis by bronchial web made two separate orifices before procedure, (B) right bronchus after removal of the web

A 4‐year‐old boy with anamneses of seasonal allergy, which was predominant in the spring and occasional cough without other comorbidities was referred to the emergency department in Shiraz following sudden cough and dyspnea. The patient underwent bronchoscopy due to foreign body aspiration suspicious. A little plastic toy with a length of 8‐mm stocked in the right main bronchus and was extracted along through the rigid bronchoscopy. Although Bronchoscopy revealed that the trachea and bronchi of the left lung were normal, severe inflammation was reported in the right bronchus possibly due to foreign body irritation. In order not to heal the cough and other symptoms, the patient was referred to several doctors who suggested to be treated as childhood asthma, but the symptoms did not remarkably improve. After 5 months of persistent symptoms, especially cough and exertional dyspnea, the patient was referred to our institution for more evaluation. His physical examination was unremarkable without audible wheezing or stridor. Results of a routine hematological panel and the chest x‐ray upon admission were interpreted as normal (Figure 1). The chest CT scan showed a partial obstruction in the main right bronchus (Figure 2). For more evaluation, a pulmonary 3‐dimensional constructed CT scan was conducted which revealed a narrowing structure at the third‐thoracic (T3) vertebrae bone level without any mass, adenopathy, or vascular sling formation in the mediastinum (Figure 3). The patient underwent bronchoscopy and a string‐like, pallor structure stretching across the main right bronchial lumen obstructed the mainstream of the lumen with just a 2 mm opening was (Figure 4). No additional anomalies of the trachea, carina, vocal cords, or other organs were found. The lesion was partially removed using knife during the bronchoscopy procedure. Removal of the lesion by knife, which was easily broken, revealed a fibrous string without bronchial glands or cartilage diagnosed as a “bronchial web” (Figure 4). No additional anomalies of the trachea, carina, vocal cords, or other organs were found. The lesion was partially removed using a knife during the bronchoscopy procedure. The lesion easily removed by Kinfe during bronchoscopy has been sent for pathological evaluation that revealed a fibrous string without bronchial glands or cartilage diagnosed as a “bronchial web”. After 7 days, we conducted the next flexible bronchoscopy to remove the remained section of the bronchial web and evaluate whether granulation tissue developed or not.

## 3 DISCUSSION

A bronchial web is formed by a thin layer of membranous tissue containing small holes that cause the bronchial lumen to narrow leading to partial airway obstruction. The etiology of the lesion is unknown. If such webs result in complete obstruction, they will be fatal unless treated at birth. The literature about which published on this topic revealed that most bronchial cases were congenital by nature and similar to our finding, the anatomical occurrence was mostly in the right bronchial site.^2^, ^3^, ^4^ Most of these cases went initially unrecognized due to the nonspecific symptoms. Similarly, our patient who initially had an episode of foreign body aspiration had been treated as asthma for frequent months due to persistent cough and dyspnea. We believe that the appearance of clinical symptoms just after the foreign body aspiration was due to inflammation reaction to the foreign body at the bronchial website, which led to exacerbating the partial lumen narrowing. In other meaning, the worsening of his symptoms probably was associated with the progression of the size of the stenosis. Our case demonstrated the need for bronchoscopy evaluation due to persistent symptoms not ameliorated by previous treatment. Diagnostic bronchoscopy is essential for the diagnosis of congenital bronchial web and also for acquired cases. Meanwhile, in recent years, tendencies to spiral pulmonary 3D‐CT scan and reconstructed virtual bronchoscopy navigation as a diagnostic option have increased.^5^ By this imaging, the internal layer of the airway's lumen and also neighboring structures of the external lumen such as fistula could be inspected.^6^ By evaluation of the primary chest x‐ray in our patient, it was not plausible to suspect bronchial narrowing (Figure 1); however, the findings of bronchial stenosis were remarkably evident in the pulmonary 3D CT scan. Moreover, as the internal aspect of bronchial lumen images was constructed by virtual bronchoscopy using the pulmonary CT‐scan images, the findings suggested web‐shaped stenosis in the right bronchus. Virtual bronchoscopy is less effective for the dynamic imaging inspection or evaluation of mucosal formations by the colorful tonnage compared with bronchoscopy; meanwhile, the virtual bronchoscopy advantage is that the bronchial lumen does not get invaded directly by the bronchoscope instrument; hence the possible injury to high‐risk lesions is prevented. Therefore, if a fixed and predominant stenotic structure is doubted at a level where bronchoscopy is hard to utilize, primary assessment using 3D pulmonary CT scan and virtual bronchoscopy could be helpful.^5^, ^7^ Finally, the interventional lesion removal using a knife during two stages of flexible bronchoscopy at one‐week intervals has not been previously reported for improvement of these lesions. Due to the complete remission of clinical symptoms and no procedure‐related side effects, this technique is found to be a safe and effective way to treat airway obstruction following bronchial web.

## 4 CONCLUSION

Herein, we described a misdiagnosed case of a child with prolonged cough and dyspnea who was ultimately diagnosed with bronchial web after evaluation via pulmonary 3D‐CT scan and treated by interventional bronchoscopy. Bronchial stenosis due to web structures should be considered in prolonged asthma‐like symptoms, and assessment based on using a 3D pulmonary CT scan, virtual bronchoscopy, and following bronchoscopy as ultimate diagnostic and treatment options could be advantageous.

## AUTHOR CONTRIBUTIONS

**Mahsa Mirzandeh del** was involved in the management of patient, conceptualization, literature review, and initial manuscript preparation. **Atefeh Abedini** was involved in the management of patient and participated in visualization and final editing. **Siamak Afaghi** and **Fatemehsadat Rahimi** contributed to writing, visualization, and draft preparation. **Arda Kiani** was project administration, involved in management of patient, reviewed the manuscript, and had final approval.

## FUNDING INFORMATION

There is no funding to present the essay.

## CONFLICT OF INTEREST

No conflict of interest is declared by the authors.

## ETHICAL APPROVAL

The authors all declare that this manuscript is not published or under consideration in other journals.

## CONSENT

Written informed consent has been acquired from the patient parents to publish this study according to the journal's patient consent policy. Moreover, the authors all declare that patients' confidentiality has been respected.

## Data Availability

The data supporting the findings of the study such as electronic medical reports and full video of bronchoscopy procedures are available upon request from the corresponding author.
